# Prevalence of Bombay blood group in a tertiary care hospital, Andhra Pradesh, India

**DOI:** 10.4103/0973-6247.76006

**Published:** 2011-01

**Authors:** Anju Verma, K. Geetha Vani, I. S. Chaitanya Kumar, D.S. Jothi Bai

**Affiliations:** Department of Transfusion Medicine, Sri Venkateswara Institute of Medical Sciences, Tirupati, Andhra Pradesh, India

Sir,

The prevalence of Bombay blood group (Oh Phenotype) in Andhra Pradesh state, South India, is not precisely known. Reported prevalence in the adjoining states of Tamil Nadu and Karnataka is 0.004[1] and 0.005%,[2] respectively. Two recent population-based surveys in Chittoor district of Andhra Pradesh on ABO blood groups[3,4] do not even mention about this rare phenotype.

Hence, the authors from a tertiary care hospital in Andhra Pradesh, that is Sri Venkateswara Institute of Medical Sciences (SVIMS), Tirupati, in their investigation covering urban as well as rural population, screened at its blood bank, the Recipients and Donor subjects over a period of one and a half years to determine the prevalence of Bombay blood group. The relatives of the index cases were also included in the study.

The ABO and Rh-D typing were performed as per the AABB Technical Manual 16th Edition, 2008. Both cell and serum grouping were done. Red cell typing was done with commercial antisera and serum grouping was done using known cells from pooled blood units. All blood samples showing “O” group were tested with commercial anti-H lectin of Ulex europeus.

Saliva samples from all Bombay group persons were tested for ABO antigens by hemagglutination inhibition test. History of any consanguinity in the parents of Bombay group subjects was recorded. Analyzing the results of 26,638 study subjects showed that the most common group was “O” group (40.21%); 13 Oh phenotypes (0.048%) were detected - 7 males and 6 females. Among these 13 Oh phenotypes, only 3 were Rh-D negative. Consanguinity among parents was observed in 10 cases (77%). Bombay phenotype was ascertained by the absence of H antigen and the non-secretor status.

To conclude, while the data on frequency of ABO groups is in concurrence with the published data, the prevalence of Bombay blood group in a mixed population covering urban and rural areas of Chittoor district, Andhra Pradesh, was found to be higher that is, 0.05% compared with other parts of South India. Consanguinity appears to be an important, preventable risk factor.

Random population surveys are needed in this aspect.
